# A case of opioid-induced rigidity requiring naloxone administration at the time of anesthesia emergence

**DOI:** 10.1186/s40981-024-00732-1

**Published:** 2024-08-01

**Authors:** Ryohei Fukasawa, Ayumi Oishi, Chiaki Nemoto, Satoki Inoue

**Affiliations:** 1 Department of Anesthesiology, Fukushima Medical University, 1 Hikarigaoka, Fukushima, 960-1295 Japan; 2 Department of Anesthesiology, Ohara General Hospital, 6-1 Ohomachi, Fukushima, 960-8611 Japan

**Keywords:** Opioid-induced rigidity, Anesthetic emergence agitation, Fentanyl, Naloxone

## Abstract

**Background:**

Opioid-induced rigidity is typically observed during rapid administration of fentanyl. Herein, we present a case in which rigidity occurred after reversal of rocuronium during emergence from anesthesia.

**Case presentation:**

A 73-year-old man underwent video-assisted partial lung resection. General anesthesia was induced with propofol, remimazolam, remifentanil, and rocuronium. Fentanyl was administered early during anesthesia. The surgery was completed without complications, and sugammadex sodium was administered for rocuronium reversal. The patient became agitated, but spontaneous breathing was maintained; therefore, the intratracheal tube was removed after the administration of flumazenil. The patient developed stiffness in the neck and jaw muscles along with remarkable skeletal muscle contractions. Dramatic improvement was observed immediately after administration of naloxone.

**Conclusions:**

Even as the simulated effect site concentration of fentanyl decreases during anesthesia emergence, opioid-induced rigidity may still occur. Rapid reversal of remimazolam by flumazenil might have contributed to the rigidity in this case.

## Background

Opioid-induced rigidity is typically observed during rapid administration of fentanyl, especially in older patients [1, 2]. Most cases develop during the induction of anesthesia [3]. In some cases, however, opioid-induced rigidity occurs even at the time of anesthesia emergence or during sedation in the intensive care unit [4–6]. The condition is characterized by rigidity of the trunk, neck, and jaw muscles [2, 7]. These symptoms can result in ventilation difficulties and may progress to life-threatening hypoxia.

We experienced a case of opioid-induced rigidity that became apparent after rocuronium reversal at emergence from anesthesia.

## Case presentation

A 73-year-old man (height, 167 cm; weight, 62 kg) with lung cancer was scheduled for video-assisted partial resection of the right lower lung lobe. He had developed a cerebral infarction 4 years previously and began taking 100 mg of aspirin daily at that time. Additionally, he had undergone burr hole craniectomy for treatment of a chronic subdural hematoma 2 years previously. Preoperative magnetic resonance imaging indicated chronic ischemic changes and diffuse microhemorrhage in the substantia alba. He had no history of taking any central nervous system agents. Preoperative blood examinations indicated no abnormalities.

**Fig. 1:**
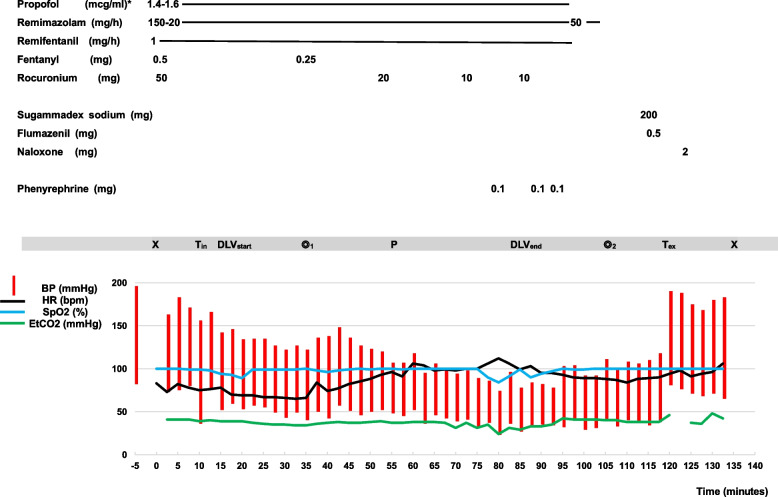
Anesthetic record. The figure shows the time course of anesthetic administration and changes in vital signs. The horizontal axis indicates the time course (minutes), and the vertical axis indicates the heart rate (bpm), saturation of percutaneous oxygen (SpO_2_; %), blood pressure (BP; mmHg), and end-tidal carbon dioxide (ETCO_2_; mmHg). *Propofol was administered using a target-controlled infusion system (Diprifusor; AstraZeneca, Cambridge, UK), with dosage units of propofol indicated by the simulated effect site concentration. The anesthetic record began at the first measurement of blood pressure (time 0), and we began administering the anesthetic agents 5 min later. The anesthesia time was 2 h 12 min, and the operation time was 1 h 10 min. X, beginning of anesthesia; ◎_1_, beginning of operation; DLV_start_, initiation of differential lung ventilation; DLV_end_, termination of differential lung ventilation; ◎_2_, end of operation; T_in_, intubation with double-lumen tube; T_ex_, tracheal extubation

General anesthesia was induced with propofol, remimazolam, and remifentanil. Approximately 1 to 2 min after administration of the anesthetic agents, we confirmed the loss of verbal commands and the eyelash reflex. We then administered 50 mg rocuronium, followed shortly by 0.5 mg fentanyl. Additionally, 0.25 mg fentanyl was administered soon after the operation began. Intraoperatively, propofol was administered at a simulated effect site concentration of 1.6 µg/mL via a target-controlled infusion system (Diprifusor; AstraZeneca, Cambridge, UK) along with concurrent infusion of remimazolam at 20 mg/h. The time course of anesthetic management and hemodynamic changes is shown in Fig. 1.

The surgery was uneventfully completed, and sugammadex sodium was administered for rocuronium reversal. The train-of-four ratio was 0.54 before the administration of sugammadex sodium, and it recovered to 1.01 soon after the reversal of rocuronium. Spontaneous respiration and the pharyngolaryngeal reflex were soon observed, but the patient became agitated. The patient was still expected to emerge from anesthesia; therefore, 0.5 mg of flumazenil was administered and the trachea was extubated. Spontaneous breathing was maintained at 10–12 breaths/min, and the SpO_2_ was 100% under oxygen flow of 4 L/min. End-tidal carbon dioxide was confirmed. Meanwhile, the patient developed stiffness in the neck and jaw muscles; remarkable skeletal muscle contraction, especially in the chest and abdomen; and forceful extension of the extremities. Although he was unable to follow visual cues, spontaneous breathing was maintained, and we continued to observe the patient. However, because these forceful muscle contractions did not resolve, we administered 0.2 mg of naloxone. Then, dramatic improvement was observed, and the patient was able to follow verbal commands. He was transferred to the postoperative unit and showed no neurological symptoms thereafter. For postoperative pain management, 1000 mg of acetaminophen was administered at the end of the surgery and the following morning, and 60 mg of oral loxoprofen sodium hydrate was initiated 6 h postoperatively and continued every 8 h. He was discharged from the hospital on postoperative day 5 with no complications.

## Discussion

Fentanyl-induced rigidity is typically reported in association with high doses of fentanyl (generally > 17 mcg/kg) [8]. However, it is also known to occur even with small boluses of 100 mcg fentanyl [1]. Considering that naloxone administration immediately relieved the muscle rigidity in our patient, it is reasonable to infer that his muscle rigidity was opioid-induced.

Opioid-induced rigidity, especially during anesthetic induction, is usually characterized by episodic breath-holding spells, tense abdominal muscles, a firmly locked jaw, and stiff extremities. Hypoxia and hypertension can also be observed during these episodes [6]. In the present case, opioid-induced rigidity upon anesthesia emergence was apparent after the administration of sugammadex sodium. Unlike typical opioid-induced rigidity, the patient’s spontaneous breathing was preserved; this made it difficult to differentiate between anesthetic emergence agitation and opioid-induced rigidity at that time. Breath-holding spells caused by opioid-induced rigidity are a manifestation of prolonged chest wall skeletal muscle contraction, which leads to decreased chest wall compliance and thus ineffective assisted and spontaneous ventilation. Even bag-valve-mask ventilation can be difficult because of high resistance [6]. These breath-holding spells caused by opioid-induced rigidity are also considered to be a result of upper airway obstruction due to glottic closure [9, 10]. From this perspective, respiration is possible if the trachea is secured by an intratracheal tube. However, in our patient, spontaneous breathing was preserved even after tracheal extubation; however, he developed tense abdominal muscles, a firmly locked jaw, and stiff extremities, and these symptoms were immediately reversed by naloxone administration.

In general, the trigger of opioid-induced rigidity is the rapid administration of a large amount of fentanyl. In our case, we administered a relatively large amount of fentanyl before the operation, but rocuronium was administered soon after fentanyl administration. Therefore, even if opioid-induced rigidity had developed, it could have been masked by the muscle relaxant effect and gone unrecognized. The opioid-induced rigidity observed at anesthesia emergence in this case manifested with sugammadex sodium administration. Our case illustrates that sustained opioid-induced rigidity, which may be masked by rocuronium, can manifest as reversal of muscular relaxation.

Roy and Fortier [4] reported a case of opioid-induced rigidity at the time of anesthesia emergence. Their patient received intermittent administration of fentanyl to a total of 0.5 mg, and termination of spontaneous breathing was thought to be largely responsible for glottic closure [4]. Even though we administered a larger amount of fentanyl than in their case, spontaneous breathing was preserved even after tracheal extubation. In our patient, the effect site concentration of fentanyl before naloxone administration was estimated to be 1.46 ng/mL using the Shafer model in the Tivatrainer simulation program (Version 8, Build 5) (Gutta B.V., EuroSIVA, Amsterdam, The Netherlands). Although the estimated effect site concentration of fentanyl provided an acceptable level of postoperative analgesia [11], rigidity may still occur even at low doses. Therefore, it may be prudent to refrain from administering potentially excessive opioids.

Roy and Fortier [4] speculated that the concurrent use of venlafaxine, which affects norepinephrine and serotonin levels, may have contributed to the rigidity in their patient. In our patient, remimazolam, an ultrashort-acting benzodiazepine [12], was administered in conjunction with propofol. Benzodiazepines are known to alleviate opioid-induced rigidity, and their antagonist flumazenil can counteract this beneficial effect [13]. Therefore, the rapid reversal of remimazolam by flumazenil might have played a role in the opioid-induced rigidity observed in our case.

In our patient, rigidity became apparent after the reversal of the muscle relaxant and was difficult to distinguish from agitation at that time. Nevertheless, oxygenation and ventilation were maintained, and flumazenil was administered with the expectation of further recovery of consciousness. Considering that rigidity can be accelerated by the administration of flumazenil, clinicians should refrain from routinely administering flumazenil. Most cases of opioid-induced muscle rigidity present with ventilatory failure due to glottic closure; therefore, our patient should have been extubated after he was fully awake, and naloxone should have been administered if rigidity was suspected before extubation.

Caution is needed because opioid-induced rigidity may occur even when spontaneous breathing is maintained. Fortunately, even after our patient’s trachea was extubated, his condition did not become critical because the glottis was not closed. However, considering that most cases of opioid-induced muscle rigidity involve glottic closure, our patient should have been extubated after resolution of the muscle rigidity by naloxone.

Both remifentanil and fentanyl can cause rigidity. Although our patient’s effect site concentration of remifentanil was very low when we observed the rigidity, a rapid decrease in the remifentanil concentration from a high level can cause withdrawal symptoms. Instead of administering relatively large doses of opioids, we should have initially used regional analgesic techniques to achieve opioid-sparing anesthesia [14]. Peripheral nerve block may help reduce opioid consumption and potentially avoid opioid-related complications.

Loss of subcortical inhibitory activity may be a common mechanism involved in the neuroexcitation characteristic of opioid-induced rigidity [4]. In the present case, the patient had a history of cerebral infarction and chronic subdural hematoma, and preoperative magnetic resonance imaging indicated chronic ischemic changes in the substantia alba. These comorbidities might have been involved in the development of opioid-induced rigidity. Our patient may have been exhibiting signs indicative of a risk of opioid-induced rigidity, but this is only speculation at this stage.

Considering his comorbidities, our patient might have also been at high risk of postoperative cognitive decline, and we should have performed intraoperative electroencephalographic monitoring. Additionally, considering his postoperative cognitive function, peripheral nerve blocks should have been used to reduce opioid consumption [15].

In summary, we experienced a case of atypical opioid-induced rigidity in which spontaneous breathing was preserved. The opioid-induced rigidity upon emergence from anesthesia was apparent after sugammadex sodium administration, and it was reversed by naloxone administration. Even as the simulated effect site concentration of fentanyl decreases during anesthesia emergence, opioid-induced rigidity may still occur. We consider that the rapid remimazolam reversal by flumazenil might have contributed to the rigidity in this case.

## Data Availability

Not applicable.
